# Supplementary material for: The effect of evocalcet on vagus nerve activity of the gastrointestinal tract in miniature pigs
Source: PLoS One. 2021 Jan 22;16(1):e0245785. doi: 10.1371/journal.pone.0245785 (PMC7822337; doi:10.1371/journal.pone.0245785)
Supplement: Attachment — Submitted filename: Review 2020 PONE-D-20-16625.doc [file pone.0245785.s007.doc]

Manuscript Number: PONE-D-20-16625

The effect of evocalcet on vagus nerve stimulation of the gastrointestinal tract in　miniature pigs

The authors analyzed the incidences of emetic symptoms induced by the administrations of cinacalcet or evocalcet in miniature pigs. Cinacalcet induced the symptoms dose-dependently, however, evocalcet did no symptom. They also examined the vagus nerve stimulation of the gastrointestinal tract by these two calcimimetics, and found that action potential of afferent vagus induced the symptom.

The experiments are well-planned and the results themselves are well-understood. Cisplatin, employed as active placebo to compare the effects of emetic symptoms and vagus nerve stimulation to calcimimetics, made their results very clear.

Although the authors concluded the emetic symptoms were evoked by CaR stimulation in stomach, the evidences were still weak, which they mentioned in the discussion. Actually hypercalcemia caused by primary hyperparathyroidism evokes many symptoms including gastrointestinal symptoms such as nausea/vomiting. These clinical evidences should be disucussed.

Page 3, line 36 “critical” is too strong, milder expressions would be suitable for this sentence.

Page 3, line 43 “higher bioavailability” needs references

Page 3, line 45 “no such effect” needs references.

Page 3, line 46 “in common marmosets” needs references.

Page 4, line 52 Why used miniature pigs? Kindly give an explanation and references.

Page 4, line 65 The expression “strict accordance” is wired. delete “strict.”

Page 5, line 77 The expression “overanesthesia” is wired. “Overanesthesia” may kill an animal.

Table 1, table 2, table 3 Kindly give P values in these tables.

Figure 3, figure 5 The time-course changes in these compound were significant?

Figure legends of fig 5

“The efferent vagus nerve” should be “The afferent vagus nerve.”
