# Supplementary material for: The effect of evocalcet on vagus nerve activity of the gastrointestinal tract in miniature pigs
Source: PLoS One. 2021 Jan 22;16(1):e0245785. doi: 10.1371/journal.pone.0245785 (PMC7822337; doi:10.1371/journal.pone.0245785)
Supplement: Attachment — Submitted filename: Response to Reviewers.docx [file pone.0245785.s008.docx]

Dear Julia Robinson

Senior Editor

PLOS ONE

Title: The effect of evocalcet on vagus nerve stimulation of the gastrointestinal tract in miniature pigs

Authors: Shin Tokunaga and myself.

We thank very much for the carefully reviewing our manuscript and the fruitful suggestions, especially for suggesting better terms and sentences concerning our manuscript. We found the comments of the reviewers most helpful and thus revised our manuscript according to the suggestions of the reviewers. We enclose revised versions of the manuscript, both with highlights of all changes and with unmarked changes. We also include a letter of our itemized responses to the Editor’s and Reviewer’s comments. We hope that this revised manuscript is now acceptable for publication in PLOS ONE.

Thank you again for considering our manuscript. We are looking forward to hearing from you soon.

Respectfully yours,

Takehisa Kawata, PhD

Nephrology Research Laboratories, Nephrology R&D Unit, R&D Division

Kyowa Kirin Co., Ltd

1188, Shimotogari, Nagaizumi-cho, Sunto-gun, Shizuoka, 411-8731, Japan

Phone: +81-80-1160-0951

e-mail: [takehisa.kawata.kk@kyowakirin.com](mailto:takehisa.kawata.kk@kyowakirin.com)

**Reviewer #1:**

The authors analyzed the incidences of emetic symptoms induced by the administrations of cinacalcet or evocalcet in miniature pigs. Cinacalcet induced the symptoms dose-dependently, however, evocalcet did no symptom. They also examined the vagus nerve stimulation of the gastrointestinal tract by these two calcimimetics, and found that action potential of afferent vagus induced the symptom.

The experiments are well-planned and the results themselves are well-understood. Cisplatin, employed as active placebo to compare the effects of emetic symptoms and vagus nerve stimulation to calcimimetics, made their results very clear.

Although the authors concluded the emetic symptoms were evoked by CaR stimulation in stomach, the evidences were still weak, which they mentioned in the discussion. Actually hypercalcemia caused by primary hyperparathyroidism evokes many symptoms including gastrointestinal symptoms such as nausea/vomiting. These clinical evidences should be discussed.

***Response:*** *Thank you for your helpful suggestions. As you pointed out, we added the sentence and the following references.*

*Page 14, line 222-227, “Although it is difficult to elucidate the mechanism of nausea and vomiting induced by calcimimetics, GI symptoms associated with hypercalcemia has been reported in various reports [19-21], which is an important knowledge for understanding. For example, it was reported the GI symptoms (anorexia, nausea, vomiting, abdominal pain, and constipation) occurred in 66% of primary hyperparathyroidism patients with hypercalcemia [22].”*

*19.	Eto S. Hypercalcemia in malignancy. Rinsho Byori. 1994;42:943-951. PMID: 7967119*

*20. Kasperk C. Hypercalcemic crisis and hypocalcemic tetany. Internist (Berl). 2017;58:1029-1036. doi: 10.1007/s00108-017-0311-3 PMID: 28835974*

*21. Taylor DM, Date PA, Ugoni A, Smith JL, Spencer WS, de Tonnerre EJ, et al. Risk variables associated with abnormal calcium, magnesium and phosphate levels among emergency department patients. Emerg Med Australas. 2020;32:303-312. doi: 10.1111/1742-6723.13411 PMID: 31847050*

*22. Aresta C, Passeri E, Corbetta S. Symptomatic hypercalcemia in patients with primary hyperparathyroidism is associated with severity of disease, polypharmacy, and comorbidity. Int J Endocrinol. 2019;2019:7617254. doi: 10.1155/2019/7617254 PMID: 32082374*

Page 3, line 36 “critical” is too strong, milder expressions would be suitable for this sentence.

***Response:*** *Thanks for your fruitful advice. We have changed the sentence as follows,*

*Page 3, line 35-37, “However, cinacalcet has adverse effects in the gastrointestinal (GI) tract [8], including nausea and vomiting.”*

Page 3, line 43 “higher bioavailability” needs references

***Response:*** *Thank you for your helpful suggestions. As you pointed out, the following references have been added to the relevant sentence.*

*13.	Tokunaga S, Endo Y, Kawata T. Pharmacological and clinical profiles of a novel calcimimetic, evocalcet (ORKEDIA^®^). Nihon Yakurigaku Zasshi. 2019;154:35-43. doi: 10.1254/fpj.154.35 PMID: 31308348*

Page 3, line 45 “no such effect” needs references.

***Response:*** *Thank you for your helpful suggestions. As you pointed out, the following references have been added to the relevant sentence.*

*11.	Kawata T, Tokunaga S, Murai M, Masuda N, Haruyama W, Shoukei Y, et al. A novel calcimimetic agent, evocalcet (MT-4580/KHK7580), suppresses the parathyroid cell function with little effect on the gastrointestinal tract or CYP isozymes in vivo and in vitro. PLoS One. 2018;13:e0195316. doi: 10.1371/journal.pone.0195316 PMID: 29614098*

Page 3, line 46 “in common marmosets” needs references.

***Response:*** *Thank you for your helpful suggestions. As you pointed out, the following references have been added to the relevant sentence.*

*11.	Kawata T, Tokunaga S, Murai M, Masuda N, Haruyama W, Shoukei Y, et al. A novel calcimimetic agent, evocalcet (MT-4580/KHK7580), suppresses the parathyroid cell function with little effect on the gastrointestinal tract or CYP isozymes in vivo and in vitro. PLoS One. 2018;13:e0195316. doi: 10.1371/journal.pone.0195316 PMID: 29614098*

Page 4, line 52 Why used miniature pigs? Kindly give an explanation and references.

***Response:*** *Thank you for your helpful suggestions. As you pointed out, we added a sentence and the following references.*

*Page 4, line 54-57, “Pigs are similar to humans in eating habits, so the structure of the digestive tract is expected to be physiologically similar to humans. [15, 16]. Since the anticancer drug cisplatin also induces vomiting in pigs [17], it is considered a suitable animal for emesis and anti-emetic studies.”*

*15.	Kararli TT. Comparison of the gastrointestinal anatomy, physiology, and biochemistry of humans and commonly used laboratory animals. Biopharm Drug Dispos. 1995;16:351-380. doi: 10.1002/bdd.2510160502 PMID: 8527686*

*16.	Vodicka P, Smetana K Jr, Dvoránková B, Emerick T, Xu YZ, Ourednik J, et al. The miniature pig as an animal model in biomedical research. Ann N Y Acad Sci. 2005;1049:161-71. doi: 10.1196/annals.1334.015 PMID: 15965115*

*17.	Milano S, Blower P, Romain D, Grélot L. The piglet as a suitable animal model for studying the delayed phase of cisplatin-induced emesis. J Pharmacol Exp Ther. 1995;274:951-961. PMID: 7636759*

Page 4, line 65 The expression “strict accordance” is wired. delete “strict.”

***Response:*** *Thank you for your fruitful advice. We have changed the sentence as follows,*

*Page 4, line 68-69, “All animal studies were carried out in accordance with the Standards for Proper Conduct of Animal Experiments at Kyowa Kirin Co., Ltd.”*

Page 5, line 77 The expression “overanesthesia ” is wired. “Overanesthesia” may kill an animal.

***Response:*** *Thank you for your comments. This section describes euthanasia of animals after the end of the study. Since the purpose was euthanasia, I used the expression "overanesthesia".*

Table 1, table 2, table 3 Kindly give P values in these tables.

***Response:*** *Thank you for your fruitful advice. As you pointed out, the p values are listed in Tables. Due to the large variability, dose dependence could not be mentioned. Therefore, it was added the sentence as follows,*

*Page 8, line 140-141, “However, due to the large variability of individual values, dose dependency could not be confirmed.”*

Figure 3, figure 5 The time-course changes in these compound were significant?

***Response:*** *Thank you for your comments. Vagus nerve action potentials vary in time and among individuals. The smaller the evaluation time, the greater the variance, and it is considered difficult to evaluate the effects of test articles. Therefore, statistical analysis was not performed on the time course.*

Figure legends of fig 5

“The efferent vagus nerve” should be “The afferent vagus nerve.”

***Response:*** *Thank you for pointing out the mistake. As you pointed out, we have changed the sentence as follows,*

*Page 12, line 189, “Fig 5. The afferent vagus nerve action potential rate of change at each time points.”*

**Reviewer #2:**

An interesting study and my comments are minor.

1. Title: Suggest changing to "vagal nerve activity" as you are studying the effects of the drugs on vagal nerve activity "at rest" not during stimulation

***Response:*** *Thank you for your fruitful suggestion. We have changed the title as follows,*

*Page 1, line 1, “The effect of evocalcet on vagus nerve activity of the gastrointestinal tract in miniature pigs.”*

2. Method: Your observations of vomiting-like behaviours includes vomiting. Better to say that you quantified the numbers of vomits (ie. oral expulsion of gastric contents), dry retching and additional behaviours associated with vomiting.

***Response:*** *Thank you for your comment. As you pointed out, we observed the tongue licking and retching as a vomiting-related behavior. These number of measurements are described on page 6, line 98-100.*

3. When compounds were administered intragastrically was this via the esophagus or an incision in the stomach?

***Response:*** *Thank you for your comment. After the electrode was connected to an amplifier, it was confirmed that the number of spikes in the vagus nerve was stable. The compounds were administered after the stabilization of spikes in vagus nerve. As you pointed out, we added the sentence as follows,*

*Page 7, line 117-118, “Cisplatin was intravenously administered, and evocalcet or cinacalcet were intragastrically administered to each drug treatment group after the stabilization of spikes in vagus nerve.”*

4. What were the statistical significance values for the initial serum PTH/calcium measurements?

***Response:*** *Thank you for your fruitful advice. As you pointed out, the p values are listed in Tables.*

5. Elsewhere, when you talk about differences which tended to occur but which were not statistically significant, give the P value.

***Response:*** *Thank you for your comments. As you pointed out, the p value was added to the description of the result that there was no significant difference between Vehicle-evocalcet and evocalcet-cinacalcet in the afferent vagus nerve action potentials.*

*Page 11, line 172-Page 12, line 174, “Although the total action potential in the evocalcet group was increased compared to that in the vehicle group, it was not significant (p=0.536) and was lower than in the cinacalcet group. However, the total afferent action potentials of the cinacalcet and evocalcet groups did not differ to a statistically significant extent (p=0.587).”*

6. Since there are other ways of causing vomiting, apart from vagal nerve activation (eg. by stimuli in the blood activating neurons in the area postrema), it would be good to say that you focussed on the vagus and not this other route because of the low oral bioavailability of cinacalcet. In addition, since the authors drug has good oral bioavailability (and therefore likely to access the area postrema), the absence of vomiting could be used to add further support to the authors argument that it is the retention of cinacalcet within the upper GI tract that leads to the GI side effects

***Response:*** *Thank you for your comment.*

**Reviewer #3:**

Authors clarified the mechanism of GI tract adverse effects of cinacalcet to measure the action potential of the vagus nerve after oral administration in miniature pigs and found the significant difference of the afferent nerve between cinacalcet and Evocalcet, a novel calcimimetic agent.

1) Based on these findings, does high concentration of calcium ion increase action potential of the afferent vagus nerve and induce vomiting through the CaR stimulation in the GI tract? Please discuss.

***Response:*** *Thank you for your comments. Although the effect of hypercalcemia on the afferent vagal nerve through CaR is unknown, there are several reports of GI symptoms associated with hypercalcemia. As you pointed out, we added a sentence and the following references.*

*Page 14, line 222-227, “Although it is difficult to elucidate the mechanism of nausea and vomiting induced by calcimimetics, GI symptoms associated with hypercalcemia has been reported in various reports [19-21], which is an important knowledge for understanding. For example, it was reported the GI symptoms (anorexia, nausea, vomiting, abdominal pain, and constipation) occurred in 66% of primary hyperparathyroidism patients with hypercalcemia [22].”*

*19.	Eto S. Hypercalcemia in malignancy. Rinsho Byori. 1994;42:943-951. PMID: 7967119*

*20.	Kasperk C. Hypercalcemic crisis and hypocalcemic tetany. Internist (Berl). 2017;58:1029-1036. doi: 10.1007/s00108-017-0311-3 PMID: 28835974*

*21. Taylor DM, Date PA, Ugoni A, Smith JL, Spencer WS, de Tonnerre EJ, et al. Risk variables associated with abnormal calcium, magnesium and phosphate levels among emergency department patients. Emerg Med Australas. 2020;32:303-312. doi: 10.1111/1742-6723.13411 PMID: 31847050*

*22. Aresta C, Passeri E, Corbetta S. Symptomatic hypercalcemia in patients with primary hyperparathyroidism is associated with severity of disease, polypharmacy, and comorbidity. Int J Endocrinol. 2019;2019:7617254. doi: 10.1155/2019/7617254 PMID: 32082374*

2) In Fig 2 and Fig 4, why did authors choose 2hr treatment of the agents, instead of 0.5-1hr treatment?

***Response:*** *Thank you for your question. We examined the maximum time that vagus nerve action potentials could be measured. Since the drugs remained in the gastrointestinal tract, it was considered necessary to observe as well as possible and two hours is the maximum time that the vagus nerve action potentials can be stably measured during the life of the miniature pigs.*

3) In Fig 5, did authors compare delta value of action potentials between (Evo-Veh) and (Cin-Veh) in acute phase (10-60 min)? If you find statistically significant difference, you may add new figure and stress the difference of these two agents.

***Response:*** *Thank you for your comments. We did not compare delta value of action potentials in acute phase.*

4) In Line 45 and Line 46, the references are necessary.

***Response:*** *Thank you for your helpful suggestions. As you pointed out, the following references have been added to the relevant sentence.*

*11.	Kawata T, Tokunaga S, Murai M, Masuda N, Haruyama W, Shoukei Y, et al. A novel calcimimetic agent, evocalcet (MT-4580/KHK7580), suppresses the parathyroid cell function with little effect on the gastrointestinal tract or CYP isozymes in vivo and in vitro. PLoS One. 2018;13:e0195316. doi: 10.1371/journal.pone.0195316 PMID: 29614098*
